# Effect of Menstrual Cycle and Hormonal Contraception on Musculoskeletal Health and Performance: Protocol for a Prospective Cohort Design and Cross-Sectional Comparison

**DOI:** 10.2196/50542

**Published:** 2024-07-11

**Authors:** Sarah J Myers, Rebecca L Knight, Sophie L Wardle, Kirsty AM Waldock, Thomas J O'Leary, Richard K Jones, Paul E Muckelt, Anton Eisenhauer, Jonathan CY Tang, William D Fraser, Julie P Greeves

**Affiliations:** 1 Army Health and Performance Research Army Headquarters Andover United Kingdom; 2 Division of Surgery & Interventional Science University College London London United Kingdom; 3 School of Health and Society University of Salford Salford United Kingdom; 4 School of Health Sciences University of Southampton Southampton United Kingdom; 5 Southampton Biomedical Research Centre National Institute for Health and Care Research Southampton United Kingdom; 6 GEOMAR Helmholtz-center for Ocean Research Kiel Kiel Germany; 7 Osteolabs Kiel Germany; 8 Norwich Medical School University of East Anglia Norwich United Kingdom; 9 Clinical Biochemistry Department of Laboratory Medicine Norfolk and Norwich University Hospital NHS Foundation Trust Norwich United Kingdom; 10 Departments of Diabetes and Endocrinology Norfolk and Norwich University Hospital NHS Foundation Trust Norwich United Kingdom

**Keywords:** estrogens, oestradiol, progesterone, calcium, musculoskeletal health, hormonal contraceptive

## Abstract

**Background:**

Women of reproductive age experience cyclical variation in the female sex steroid hormones 17β-estradiol and progesterone during the menstrual cycle that is attenuated by some hormonal contraceptives. Estrogens perform a primary function in sexual development and reproduction but have nonreproductive effects on bone, muscle, and sinew tissues (ie, ligaments and tendons), which may influence injury risk and physical performance.

**Objective:**

The purpose of the study is to understand the effect of the menstrual cycle and hormonal contraceptive use on bone and calcium metabolism, and musculoskeletal health and performance.

**Methods:**

A total of 5 cohorts of physically active women (aged 18-40 years) will be recruited to participate: eumenorrheic, nonhormonal contraceptive users (n=20); combined oral contraceptive pill (COCP) users (n=20); hormonal implant users (n=20); hormonal intrauterine system users (n=20); and hormonal injection users (n=20). Participants must have been using the COCP and implant for at least 1 year and the intrauterine system and injection for at least 2 years. First-void urine samples and fasted blood samples will be collected for biochemical analysis of calcium and bone metabolism, hormones, and metabolic markers. Knee extensor and flexor strength will be measured using an isometric dynamometer, and lower limb tendon and stiffness, tone, and elasticity will be measured using a Myoton device. Functional movement will be assessed using a single-leg drop to assess the frontal plane projection angle and the qualitative assessment of single leg loading. Bone density and macro- and microstructure will be measured using ultrasound, dual-energy x-ray absorptiometry, and high-resolution peripheral quantitative computed tomography. Skeletal material properties will be estimated from reference point indentation, performed on the flat surface of the medial tibia diaphysis. Body composition will be assessed by dual-energy x-ray absorptiometry. The differences in outcome measures between the hormonal contraceptive groups will be analyzed in a one-way between-group analysis of covariance. Within the eumenorrheic group, the influence of the menstrual cycle on outcome measures will be assessed using a linear mixed effects model. Within the COCP group, differences across 2 time points will be analyzed using the paired-samples 2-tailed *t* test.

**Results:**

The research was funded in January 2020, and data collection started in January 2022, with a projected data collection completion date of August 2024. The number of participants who have consented at the point of manuscript submission is 66. It is expected that all data analysis will be completed and results published by the end of 2024.

**Conclusions:**

Understanding the effects of the menstrual cycle and hormonal contraception on musculoskeletal health and performance will inform contraceptive choices for physically active women to manage injury risk.

**Trial Registration:**

ClinicalTrials.gov NCT05587920; https://classic.clinicaltrials.gov/ct2/show/NCT05587920

**International Registered Report Identifier (IRRID):**

DERR1-10.2196/50542

## Introduction

### Background

Women of reproductive age experience cyclical changes in endogenous sex steroid hormones during the menstrual cycle. These hormones regulate and support the production, release, and fertilization of the ovum. 17β-estradiol, the predominant estrogen in women of reproductive age, is produced by the ovaries during the early stages of the menstrual cycle. Progesterone is secreted by the *corpus luteum*—the discarded sac of the ovum—after ovulation and stimulates endometrial growth in preparation for fertilization in the later stages of the menstrual cycle. Growth and release of ova are regulated by the anterior pituitary hormones—follicle-stimulating hormone and luteinizing hormone (LH)—under the influence of gonadotropin-releasing hormone [1].

Hormonal contraceptives (HCs) are made from synthetic forms of estrogens or progesterone, in some instances combined in varying concentrations. They prevent pregnancy by disrupting the release of gonadotropin-releasing hormone, suppressing ovulation, and altering the uterine lining to impair egg implantation. Consequently, the majority of HCs reduce circulating endogenous sex steroid hormones [2]. The most commonly used HC is the combined oral contraceptive pill (COCP), but long-acting reversible contraceptives (LARCs) containing progestin only, administered by subcutaneous implant, intrauterine system (IUS), or intramuscular injection, are increasingly popular methods [3]. Some studies have measured temporal patterns of sex steroid hormones during the menstrual cycle [4] and in COCP users [5,6], but data are lacking in LARC users.

HCs are also used medically to reduce or suppress menstrual bleeding in those experiencing conditions such as endometriosis [7] and by female athletes and servicewomen to delay or prevent menstrual bleeding [8,9]. However, there is growing concern that the noncontraceptive effects of estrogens on bone, muscle, and metabolism may influence injury risk and physical performance [10]. Estrogens are essential to bone health; women who experience conditions with lower estrogens, such as postmenopausal women [11] and those with functional hypothalamic amenorrhea [12], have lower bone mass compared with eumenorrheic (EUM) women. The influence of HCs on bone mass and bone stress injury risk is less clear [13], although biochemical markers of bone resorption and formation are lower in COCP users compared with EUM women [14-16]. The use of the COCP may decrease bone remodeling by a reduction in endogenous estrogens or indirectly by a synthetic estrogen-mediated reduction in *free* insulin-like growth factor I (IGF-I) [17].

Osteogenesis is the formation of new bone tissue performed by osteoblasts. Bone resorption is the process by which osteoclasts break down bone tissue, releasing calcium and other minerals into the circulatory system. Biochemical markers of bone resorption and formation reflect metabolic processes in bone, but they do not provide quantitative data on calcium status. A total of 6 naturally occurring calcium isotopes exist (^40^Ca, ^42^Ca, ^43^Ca, ^44^Ca, ^46^Ca, and ^48^Ca). Bone formation favors the uptake of lighter calcium isotopes and subsequently causes the composition of urine to shift toward an isotopically heavier state [18]. If bone resorption is the dominant metabolic process and calcium is excreted from the bone, the calcium isotopic composition of the urine becomes lighter [19]. Low estrogens increase bone resorption, but the effect of estrogen status on calcium homeostasis (ie, whether calcium is being deposited or lost from bone), measured from ^44^Ca:^42^Ca isotopes, is not known.

Traditionally, bone strength has been inferred from measures of areal bone mineral density (aBMD) with dual-energy x-ray absorptiometry (DXA). DXA is not portable and emits ionizing radiation, and an alternative ultrasound-based method may provide valid hip and spine aBMD measurements that can be used in field-based scenarios. Additionally, peripheral quantitative computed tomography (pQCT; volumetric bone mineral density and structure) and impact microindentation provide additional, and possibly superior, estimates of bone strength in vivo in young adult healthy women compared with DXA [20]. Preliminary data using pQCT in female soldiers indicate differential effects of COCP and non-HC use on tibial adaptations during arduous training [21], with implications for stress fracture risk. High-resolution pQCT (HRpQCT) offers more detailed information on bone strength based on bone microstructure [22]. Hypoestrogenic athletes have poorer bone microarchitecture and higher stress fracture risk compared with their EUM counterparts [12,23], while female military recruits taking progestin-only contraceptives experience blunted tibial adaptations to military training compared with COCP and EUM women [24].

Anterior cruciate ligament injury risk is highest mid-cycle in EUM athletes [25,26], with a lower risk in COCP users [26], suggesting a role of estrogens. In skeletal muscle, estrogens are purported to exert beneficial effects, and low estrogens status can impair muscle strength and growth [27,28]. Menstrual bleeding and HC use can influence iron status, including the prevalence of iron deficiency, through excessive iron loss or by mediating hepcidin production, an acute-phase protein that impairs iron absorption. Understanding the effects of the menstrual cycle and hormonal contraception on musculoskeletal health and performance will inform contraceptive choices for physically active women to manage injury risk.

### Aims and Hypotheses

There is limited evidence for the overarching effects of estrogen on bone and calcium metabolism and musculoskeletal performance and even fewer studies investigating the impact from chronic use of HCs. Our primary aims are to (1) investigate the effect of menstrual cycle phase on bone and calcium metabolism, and musculoskeletal health and performance in EUM, non-HC users (EUM group); and (2) investigate the effects of HC use on bone and calcium metabolism, and musculoskeletal health and function. Our secondary aims are to (1) determine the validity of an ultrasound-based method of measuring hip and spine aBMD compared with “gold standard” DXA and (2) compare calcium and bone metabolism, reproductive hormones, and musculoskeletal function between the pill phase and non-pill phase of COCP use.

This study will test the following hypotheses in 2 study designs. Menstrual cycle phase (within-groups design): (1) calcium and bone metabolism is decreased during the ovulatory phase compared with other phases; and (2) muscle strength and tissue mechanical properties differ across the menstrual cycle. HC use (between-groups design): (1) calcium and bone metabolism is higher in the implant and injection groups compared with IUS (which exerts localized effects) and EUM (ovulatory phase) groups, and lower in the COCP group; (2) estradiol and progesterone are lower in HC users compared with the EUM group during the ovulatory phase; (3) bone macro- and microstructure, muscle strength, and tissue properties are different in HC users compared with the EUM group; and (4) calcium and bone metabolism, reproductive hormones, and musculoskeletal function are different between the pill phase and non-pill phase of COCP use.

## Methods

### Recruitment

**Figure 1:**
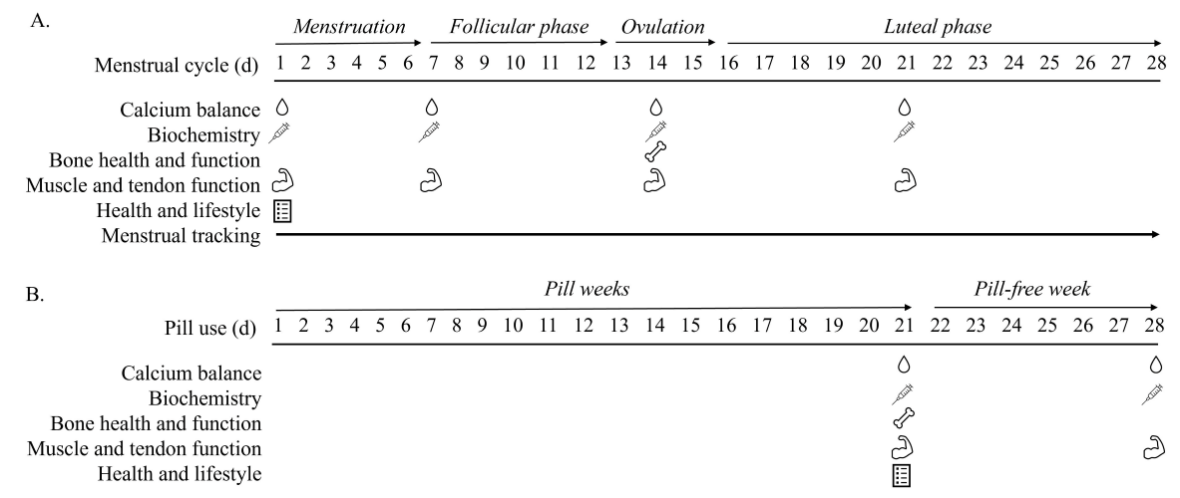
Study design schematic for (A) eumenorrheic women with an example 28-day cycle and (B) combined oral contraceptive pill users. Calcium balance represents a urine sample; biochemistry indicates a blood sample; bone health and function represents dual-energy x-ray absorptiometry scan, high-resolution peripheral quantitative computed tomography scan, and EchoLight ultrasound; muscle and tendon function represents muscle strength testing, MyotonPRO, and single-leg drop; and health and lifestyle represents questionnaires.

Biochemical markers of bone and calcium metabolism and measures of musculoskeletal health and performance will be obtained in physically active women who are EUM (n=20) or HC users taking or administered COCP (n=20), implants (n=20), IUS (n=20), or hormonal injections (n=20). Participants will be aged 18-40 years; have stable body mass (defined as no change in self-reported body mass ≥5% over the previous 3 months); have a BMI between 18 and 30 kg/m^2^; have a regular menstrual cycle of 24-35 days in length; have not been taking any HC in the last 12 months (EUM group); have used the COCP for at least 12 months (COCP group); have used the IUS continuously for at least 2 years (IUS group); have used the implant continuously for at least 12 months (implant group); or have used the hormonal injection continuously for at least 2 years (injection group). Participants will be excluded if they have diagnosed premature ovarian insufficiency; are pregnant; are less than 2 years post partum; have given birth to more than 2 children; demonstrate evidence of disordered eating (defined as ≥20 on the Eating Attitudes Test-26 items) or have a self-reported eating disorder; report menstrual disturbance (amenorrhea or oligomenorrhea: <9 menstrual cycles in previous 12 months); are a habitual smoker (defined as ≥10 cigarettes per day); are taking any medication known to affect bone or calcium metabolism (eg, treatment for thyroid disorders); have a self-declared history of heart, liver or kidney disease, diabetes or thyroid disorder; self-report a bone injury in the previous 12 months; or have a total 25-hydroxyvitamin D (25(OH)D)<30 nmol/L at prescreening. Finally, any participants not in the injection group who report a history of using the hormonal injection will also be excluded from participation. We will aim to recruit matched sample sizes; however, some HC options are less common, and the study may be unable to achieve the desired sample size. Researchers have stated an end point for data collection of August 2024, giving 2.5 years of recruitment. If any group is underpowered, the sample achieved will be presented to mitigate influences from different time periods. An overview of the study schedule is illustrated in Figure 1; proposed biochemical markers of reproductive health and bone health are listed in Table 1.

**Table 1:** Proposed biochemical measurements.

Biochemical measurements		Measurement time points, n		
		EUM^a^ group	COCP^b^ group	Other HCs^c,d^ group
**Reproductive, stress, and metabolic hormones**				
	Estradiol	4	2	1
	Progesterone	4	2	1
	Luteinizing hormone	4	2	1
	Follicle-stimulating hormone	4	2	1
	Testosterone	4	2	1
	Sex hormone–binding globulin	4	2	1
	Cortisol	4	2	1
	Cortisol-binding globulin	4	2	1
	Insulin-like growth factor I (IGF-I)	4	2	1
	IGF-binding protein 1	4	2	1
	IGF-binding protein 3	4	2	1
	Prolactin	4	2	1
	Relaxin	4	2	1
	Free thyroxine (FT4)	4	2	1
	Free triiodothyronine (FT3)	4	2	1
	Thyroid-stimulating hormone (TSH)	4	2	1
	Androstenedione	4	2	1
	Dehydroepiandrosterone sulfate	4	2	1
**Bone**				
	C-terminal telopeptide of type 1 collagen	4	2	1
	Bone–specific alkaline phosphatase	4	2	1
	Amino terminal propeptide of type 1 procollagen	4	2	1
	Osteoprotegerin	4	2	1
	Receptor activator of nuclear factor κβ ligand	4	2	1
**Iron**				
	Hepcidin-25	4	2	1
	Ferritin	4	2	1
	Soluble transferrin receptor	4	2	1
	Hemoglobin	4	2	1
**Vitamin D**				
	Total 25-hydroxyvitamin D (25(OH)D)	2	2	2
	Free total vitamin D (25(OH)D)	1	1	1
	Total 24,25-dihydroxyvitamin D	1	1	1
	1,25-Dihydroxyvitamin D	1	1	1
	Vitamin D–binding protein	1	1	1
	Intact parathyroid hormone	4	2	1
	Phosphate	4	2	1
**Calcium**				
	^44^Ca:^42^Ca ratio	4	2	1
	Albumin-adjusted calcium	4	2	1
	Ionized calcium	4	2	1

^a^EUM: eumenorrheic.

^b^COCP: combined oral contraceptive pill.

^c^HC: hormonal contraceptive.

^d^Other HCs include the hormonal injection, hormonal implant, and hormonal intrauterine system.

All participants will attend a prescreening visit to confirm eligibility and to complete familiarization with the muscle strength and functional movement testing protocols. Thereafter, the EUM group will attend the laboratory on 4 occasions, COCP users will attend the laboratory on 2 occasions, and LARC users on 1 occasion (Figure 1). The first visit for the EUM group will be scheduled on day 2 of the menstrual cycle (ie, the second day of the menstrual bleed), according to the predictions based on their cycle length (see the *Menstrual Cycle Tracking* section). As the predictions may not be exact, the first day of the menstrual bleed will be recorded, and we will accept participant testing between day 1 and day 3 of their menstrual bleed. The second visit will be scheduled at the midpoint between day 1 of the menstrual cycle and predicted ovulation. The third visit will be scheduled to coincide with ovulation, predicted from the first day of menses and then determined in *real time* from a urine test for ovulation (LH stick, Clear Blue Advanced Digital) on the days around predicated ovulation. The final visit will be scheduled between ovulation and menstruation (for a 28-day cycle, day 21) as determined by the fertility tracker. Study visits will be scheduled on days 21 (end of active pill phase) and 28 (end of pill-free week) for COCP users (Figure 1B) and at one point in time for hormonal implant, IUS, and hormonal injection users (not represented in Figure 1).

### Ethical Considerations

This study received ethical approval from the Ministry of Defence Research Ethics Committee (1042/MODREC/20) in September 2020. Participants will be provided with an information sheet at least 24 hours before consenting to participation and given a verbal description of the study. Written informed consent will be obtained at the start of the first testing session. Adverse events and reactions will be reported to the trial committee and considered by a minimum of 3 trial committee members.

All data will be handled in accordance with the project’s data management plan and in compliance with the General Data Protection Regulation. All raw data will be pseudonymized during the trial. Anonymized data will be uploaded to the active research data storage system at Nottingham Trent University for a minimum of 10 years following the completion of the study. Data access rights will be provided only to the relevant members of the research team.

### Measurements

#### Menstrual Cycle Tracking

Menstrual cycle phase and length will be monitored in the EUM group; many participants may already be using a menstrual tracking app, which will aid in cycle prediction using historical cycle length data.

Participants will be asked to report day 1 of menses, and all subsequent laboratory visits will be planned according to their reported cycle length. To verify the predicted phases of the menstrual cycle, the EUM group will be given urine dip stick tests (LH surge test) to test for ovulation between days 10 and 15 of the menstrual cycle for a 28-day cycle to ensure that testing occurs during the ovulatory phase. Women will be scheduled for testing at their *predicted* ovulation but will be *actually* tested once they also produce a positive LH test; we expect these dates to coincide. If a woman does not ovulate during the testing cycle, she will be invited to retest the following month.

#### Venous Blood and Urine Sampling

Venous blood samples will be taken in a resting state after an overnight fast. For the EUM group, blood will be drawn at 4 time points: menses (days 1-3), midfollicular phase (day 7, for women with a 28-day cycle), ovulation phase (day 14 for women with a 28-day cycle), and midluteal phase (day 21 for women with a 28-day cycle). For COCP users, samples will be obtained on day 21 of the pill cycle and the final day of the pill-free week (day 28). LARC users will be tested at a single time point. First-void urine samples will be taken at the same time points as venous blood samples.

#### Biochemical Analyses

Analysis of biochemical markers of reproductive status, bone resorption and formation, and iron status (Table 1) will be performed at the Bioanalytical Facility, University of East Anglia (Norwich, United Kingdom), according to manufacturers’ instructions and under Good Clinical and Laboratory Practice conditions. Metabolites will be analyzed using the COBAS automated platform (Roche Diagnostics) for 17β-estradiol (pmol/L), follicle-stimulating hormone (U/L), LH (U/L), procollagen type 1 N-terminal propeptide (g/L), phosphate (mmol/L), intact parathyroid hormone (pmol/L), beta carboxy-terminal cross-linking telopeptide of type 1 collagen (g/L), osteocalcin (g/L), albumin-adjusted calcium (mmol/L), IGF-I (ng/mL), prolactin (ng/mL), free thyroxine (pmol/L), thyroid-stimulating hormone (mIU/L), ferritin (g/L), soluble transferrin receptor (nmol/L), and IGF binding protein 3 (ng/mL). Liquid chromatography tandem mass spectrometry will be used for measurement of serum testosterone (nmol/L); androstenedione (nmol/L); dehydroepiandrosterone sulfate (nmol/L); cortisol (nmol/L); progesterone (nmol/L); 25(OH)D3/D2; 24,25-dihydroxyvitamin D3/D2 (nmol/L) [29]; 1,25-dihydroxyvitamin D3/D2 (pmol/L); and hepcidin-25 (ng/mL). Plate-based enzyme-linked immunosorbent assay will be used to analyze bone-specific alkaline phosphatase (U/L; MicroVue, Quidel Corp.), relaxin (pg/mL), and IGF-binding protein 1 (R&D Systems, Inc.; pg/mL). All assays will be performed with an inter- or intra-assay precision coefficient of variation (CV) of <10% across their respective assay ranges. The performance of 25(OH)D and 1,25-dihydroxyvitamin D assays is certified by the vitamin D external quality assessment scheme [30].

Hemoglobin and ionized calcium will be measured by a portable blood analyzer (iStat Alinity). Bone calcium balance will be measured in first-void urine samples, determined from the ratio of naturally occurring stable calcium isotopes (^44^Ca:^42^Ca). Total calcium and the stable calcium isotopes ^42^Ca and ^44^Ca will be analyzed using methods described elsewhere [31].

#### aBMD Measurement

Whole-body, right hip, and lumbar spine aBMD and whole-body fat mass and lean mass will be measured from a DXA scan (Lunar iDXA, GE Healthcare). Participants will be asked to remove excess jewelry and to wear shorts and a T-shirt. Scans will be performed on day 14 for the EUM group, day 21 for COCP users, and on the sole visit for LARC users.

#### Ultrasound

A nonradiating ultrasound technique (EchoLight, EchoLight S.p.a.) for measuring aBMD at the hip and lumbar spine will also be used in this study. The Royal Osteoporosis Society has recognized the technique as a clinical tool for measuring hip and spine aBMD, but there is no published research validating this technique in young, healthy adults. The site-specific aBMD readings from the ultrasound will be compared with those derived from DXA to validate the use of this equipment in future research. EchoLight ultrasound will be performed on day 14 for EUM, day 21 for COCP users, and the sole testing day for LARC users.

#### Bone Macro- and Microstructure

Bone microstructure, geometry, and volumetric density of the right tibia (distal 4% and 30% sites from the endplate) and radius will be assessed using HRpQCT (XtremeCT II, Scanco Medical AG). Trabecular and cortical volumetric bone mineral density and geometry, trabecular microarchitecture, cortical porosity, and estimated mechanical strength will be calculated for each scan. HRpQCT scans will take place on day 14 for the EUM group, day 21 for COCP users, and on the sole visit for LARC users.

#### Bone Material Properties

Reference point indentation will be performed on the flat surface of the medial tibial diaphysis using the handheld portable OsteoProbe (ActiveLife). The OsteoProbe is used to measure skeletal material mechanical properties by creating microindentations (150-260 μm depth) in the bone [32]. Reference point indentation will take place away from the main testing schedule for 1 day per month. The menstrual cycle phase and COCP status at the time of testing will be recorded.

#### Muscle and Tendon Characteristics

Measurements of muscle and tendon characteristics (see the *Muscle Strength*, *Functional Movement Testing*, and *Tissue Characteristics* sections) will be performed on every testing visit: days ~1, 7, 14, and 21 in the EUM group (equivalent to menses and midfollicular, ovulation, and midluteal phases), days 21 and 28 in the COCP group, and on the sole visit for the other HC groups.

#### Muscle Strength

Isometric and dynamic leg strength will be assessed using an isokinetic dynamometer (Biodex System 4 Pro, IPRS Mediquipe). For the isometric strength test, the participant will perform 2 submaximal warm-up reps of increasing effort followed by 3 maximal isometric contractions in extension, sustained for 5 seconds at 90° of knee flexion, with a 90-second rest period between each contraction. For the dynamic strength test, participants will complete 6 maximal repetitions in extension and flexion at 60°/s. Muscle endurance will be assessed by 15 maximal repetitions in extension and flexion at 180°/s. At least 3 minutes of rest will separate each test. Participants will be given verbal encouragement throughout the tests.

#### Functional Movement Testing

Participants will perform a single-leg drop to evaluate knee and ankle stability. From a raised platform, participants will stand on one leg with their arms crossed across their chest and “hop off” the platform, landing on the same leg. A total of 3 “hops” will be performed on each leg, with stability assessed using the qualitative assessment of single leg loading [33]. The frontal plane projection angle will be calculated by measuring the angle between the line from the proximal thigh to the midpoint of the knee joint and the line from the knee joint to the midpoint of the ankle, at the frame corresponding to maximum knee flexion [34].

#### Tissue Characteristics

Stiffness (N/m), tone (inferred from frequency; Hz), and elasticity (logarithmic decrement) of the *rectus femoris*, patellar tendon, *soleus*, *gastrocnemius*, and Achilles tendon will be measured in the rested state using digital palpation (MyotonPRO, Myoton AS). Participants will lie supine for the *rectus femoris* and patellar tendon measurements, and prone for the *soleus*, *gastrocnemius*, and Achilles tendon measurements using standardized protocols to ensure reliable recordings [35]. The MyotonPRO will be held perpendicular to the skin and 5 low-force (0.4 N) and short (15 ms) mechanical impulses applied, leading to oscillations of the underlying tissues from which stiffness, tone, and elasticity are derived. The MyotonPRO device records the CV between the 5 mechanical impulses within each set and displays this as a percentage next to each parameter. If any parameters are over a CV 3% threshold, the measurement will be repeated.

### Health and Lifestyle

#### Eating Attitudes Test 26-Items

The Eating Attitudes Test-26 item questionnaire will be completed at baseline and will be used for screening energy deficiency as a key risk factor for musculoskeletal health [36].

#### Habitual Calcium Intake

A food frequency questionnaire will be completed by participants on the final study visit to calculate habitual energy and calcium intake over the study period.

### Statistical Analyses

The required sample size was calculated in G*Power 3 based on testing the null hypotheses: (1) there will be no change in calcium balance across the menstrual cycle; and (2) there will be no difference in calcium balance between the EUM group and HC users. There are no data examining calcium balance across the menstrual cycle. We anticipate a moderate to large effect size based on reported differences in calcium balance between women with low (anorexia nervosa patients) and normal estrogen (control participants; −50, SD 116 vs 77, SD 69 mg/day; *d*=2.27) [37], circulating blood calcium between the luteal and follicular phases (9.29, SD 0.52 vs 10.08, SD 0.48 mg/dL; *d*=1.08 [38]; and 2.1, SD 0.2 vs 2.3, SD 0.2 mg/dL; *d*=1.00 [39]), and urinary calcium excretion between the luteal and follicular phases (2.1, SD 1.5 vs 3.8, SD 2.2 μM/min; *d*=0.90) [40]. We estimate that between 8 and 17 participants are required to detect a significant main effect of time for calcium balance across the menstrual cycle (measured at 7 time points) with a moderate to large effect size (eta squared 0.06-0.14), an α of 0.05, and a 1– of 0.80.

All continuous data will be checked for normality using a Shapiro-Wilk test and by visually checking for skewness and kurtosis. Markers of bone and calcium metabolism, reproductive function, and musculoskeletal health will be compared between contraceptive groups with a one-way between-group analysis of covariance (EUM [day 14] vs COCP [day 21] vs hormonal implant vs IUS vs hormonal injection groups) controlling for age, lean body mass (for bone measures only), physical activity, total 25(OH)D, and previous hormonal injection use. Categorical data from questionnaires will be analyzed using a chi-square test to test for differences between lifestyle, prior HC use, menstrual cycle history, and dietary choices between groups. Nonnormally distributed data will be tested using a Kruskal-Wallis test. Significant main effects of the group will be followed up with post hoc 2-tailed *t* tests with a Bonferroni correction (or Mann-Whitney *U* test for nonparametric data). Paired-sample 2-tailed *t* tests will be used to compare markers of bone and calcium metabolism, and reproductive and musculoskeletal function across the 2 time points in COCP users. Markers of bone and calcium metabolism, reproductive function, and muscle strength and tendon function will be compared between days in the menstrual cycle for the EUM group with a linear mixed effects model with the restricted maximum likelihood estimator to allow incorporation of incomplete data. Time will be the fixed effect (day 1 vs day 7 vs day 14 vs day 21), and a random intercept will be assigned to participants to account for the nonindependence of repeated measures. The significance of the fixed effects from each model will be determined with Sattherwaite degrees of freedom. The normality of the residuals for each model will be checked visually by plotting the residuals against the fitted values and from Q-Q plots. In the event of a significant main effect of time, pairwise comparisons with Holm-Bonferroni corrections and Kerward-Roger degrees of freedom will be used on the linear mixed effects model to identify differences between time points. A Bland-Altman plot will be used to assess the agreement between aBMD measured by ultrasound and DXA. Differences will be considered statistically significant at an α level of *P*<.05 with post hoc *P* values corrected for multiple comparisons.

## Results

The research was funded in January 2020, and data collection started in January 2022, with a projected data collection completion date of August 2024. The number of participants that have consented at the point of manuscript submission is 66. It is expected that all data analysis will be completed and results published by the end of 2024.

## Discussion

### Primary Findings

HC use is highly prevalent among servicewomen [41]. Military training is physically arduous, particularly for women [42], who experience a greater risk of musculoskeletal injuries and stress fractures than men [43]. Previous studies have shown that HC choice may inhibit skeletal adaptation to military training [24,44] and may increase stress fracture risk [45]. These studies have combined progestogen-only methods and do not report effects on muscle and soft tissue, which are limitations this study aims to address. There are limited data on the influence of contraceptives on musculoskeletal health and performance. We aim to characterize the musculoskeletal health and function of regularly menstruating women across the menstrual cycle and compare the outcomes of women using different types of HCs. These data are principally needed to understand the potential musculoskeletal injury risk (or protection) from the use of HCs in female military personnel, but the findings will have applications across wider physically active occupational groups and for female athletes and may be incorporated into public health guidance for the general population. Understanding the effects of estrogens and progesterone or progestogen on musculoskeletal health and performance will inform contraceptive choices for women and improve our understanding of the musculoskeletal risks of contraceptive use and the menstrual cycle stage.

### Strengths

Through the use of strict eligibility criteria, we will be able to control for factors that may affect the study outcomes, including age, BMI, pregnancy and postpartum status, bone injury, length of HC use, pathologies affecting hormonal functions (eg, thyroid disease), and specific HC type. This is one of the first studies of its kind to investigate such a broad range of HCs and consider all 4 phases of the menstrual cycle for physical performance assessment.

### Limitations

The cross-sectional study design prevents standardized and controlled administration (frequency and dose) of HCs in the COCP group, and the self-selection of hormonal contraception across groups may introduce selection bias. A randomized control trial design of HC interventions was not considered feasible for this research due to the length of time needed to elucidate meaningful effects, the impact on lifestyle and health care for participants, and the invasive procedures for the administration of LARCs. Functional movement—visually assessed from the location of the midpoint of the knee and ankle using video footage—may also be subject to observer bias; this bias will be minimized by using a single trained observer. Investigators will also be involved in the testing of all study participants and will not be blinded to HC groups.

### Future Directions

The research findings are expected to support the education of women in the military and policy for HC choices. The authors also anticipate identifying additional research needs for the wider impacts of long-term HC use. Investigating injury risk in association with HC use or the impact of HCs during arduous training would be particularly relevant in a military setting. These data also have important implications for exercising women in the general population. An exploration of the lasting effects post-discontinuation of HCs focused on musculoskeletal health and fertility would enhance the research in this field.

### Dissemination Policy

The trial results will be reported to the Ministry of Defence upon completion of all data collection and analysis. The evidence provided by this study will inform servicewomen of the effects of different HC options on their physical strength and performance and guide the decisions of health care professionals when prescribing HCs. With the approval of the Ministry of Defence, the results will be published in several formats, including, but not limited to, peer-reviewed journals, press releases, and conferences.

## Abbreviations

25(OH)D: hydroxyvitamin D
aBMD: areal bone mineral density
COCP: combined oral contraceptive pill
CV: coefficient of variation
DXA: dual-energy x-ray absorptiometry
EUM: eumenorrheic
HC: hormonal contraceptive
HRpQCT: high-resolution peripheral quantitative computed tomography
IGF-I: insulin-like growth factor I
IUS: intrauterine system
LARC: long-acting reversible contraceptive
LH: luteinizing hormone
pQCT: peripheral quantitative computed tomography

## Data Availability

The data sets generated during and/or analyzed during this study are not publicly available as they are held under license by The Ministry of Defence but are available from the corresponding author on reasonable request and with the permission of The Ministry of Defence.
